# Resilience and social support as protective factors against suicidal ideation among tertiary students during COVID-19: a cross-sectional study

**DOI:** 10.1186/s12889-024-19470-1

**Published:** 2024-07-19

**Authors:** Špela Selak, Nuša Crnkovič, Andrej Šorgo, Branko Gabrovec, Katarina Cesar, Mark Žmavc

**Affiliations:** 1 National Institute of Public Health, Ljubljana, Slovenia; 2 Faculty of Natural Sciences and Mathematics, University of Maribor, Maribor, Slovenia; 3 Centre for Digital Wellbeing Logout, Ljubljana, Slovenia

**Keywords:** Suicidal ideation, Depression, Resilience, Social support, Tertiary students, COVID-19 pandemic

## Abstract

**Background:**

Suicidal ideation is a depression symptom which represents a key (cognitive) component of suicidality and plays an important role in suicide risk detection, intervention, and prevention. Despite existing research showing the importance of certain factors of depression symptoms and suicidal ideation, less is known about the interaction between the various risk and protective factors. The aim of the study was to examine whether living conditions characteristics and personal circumstances during the COVID-19 pandemic predicted the presence of depression symptoms and suicidal ideation among tertiary students and whether resilience and social support can mitigate the detrimental effects of difficult life circumstances.

**Method:**

A large online cross-sectional study was conducted in March 2021 among 4,645 Slovenian tertiary students. Hierarchical multiple regression and hierarchical logistic regression methods were used to assess and compare the effect of life circumstances variables, as opposed to resilience and social support, on depression symptoms and suicidal ideation.

**Results:**

Female gender, single relationship status, living alone, a higher degree of household conflict, having a history of mental illness and chronic disease diagnosis were significant predictors of depression scores. All but gender were also predictors of suicidal ideation. Household conflict and a history of mental illness were the factors showing the strongest effect in both cases. On the other hand, social support and, in particular, resilience proved to be strong protective factors against depression symptoms and suicidal ideation. After accounting for one’s resilience and social support, the explained variance in depression scores was more than doubled, while the harmful effect of household conflict and history of mental illness significantly decreased.

**Conclusions:**

The findings stress the importance of one’s resilience and social support and explain why some people manage to maintain mental well-being despite finding themselves in difficult life circumstances, which was the case for many tertiary students during the COVID-19 pandemic. These insights may inform preventive efforts against developing suicidal ideation and may be used as support for the design and implementation of interventions for improving resilience and social support from childhood onward.

## Introduction

Suicide is a major public health challenge that puts a heavy economic, social and psychological burden on individuals, families and society. According to the World Health Organization [1], suicide accounts for more than 700,000 deaths annually. It can occur in most age groups and is the fourth leading cause of death in the age group between the ages of 15 and 29 globally [2]. Although suicide mortality has decreased by more than 30% in the last two decades in Slovenia, the suicide rates remain higher than the European average [3]. In 2021, the suicide rate in Slovenia (number of deaths by suicide per 100,000 inhabitants) was 20.5 (31.9 for men and 9.0 for women) [4].

The concept of suicidality includes a behavioral component consisting of various forms of suicidal behavior (suicide attempt, suicide) as well as a cognitive component, which consists of suicidal thoughts or ideation, suicidal intent and a suicidal plan [5]. The term suicidal ideation describes a spectrum of contemplations, wishes, and preoccupations with death and suicide [6], and is also recognized as a symptom of depression according to both DSM-V and ICD-11 [7, 8]. Most people have control over suicidal ideation and do not attempt suicide. However, the phenomenon may still play an important role in suicide risk detection, intervention, and prevention [6], as suicidal ideation was found to predict suicide attempts and deaths regardless of age [9]. Consequently, it presents one of the key measures of suicidality and is the focus of the present study.

Since suicide represents a frequent cause of death among emerging adults [2], it is important to explore the specifics of this developmental stage that may contribute to the risk of suicide. One such specific is the process of tertiary education, which comes with many benefits and major life changes (e.g., living with friends, less parental control) [10]. The COVID-19 pandemic and the associated measures implemented to prevent the spread of the infection temporarily deprived students of various benefits associated with this life period. For tertiary students, the most impactful measure was likely the closure of faculties and student dormitories, which forced many students to return to the home environment, while the educational process was transferred online. These extraordinary living conditions and study requirements affected students’ mental health and may have also contributed to depression [11–13], as well as suicidal thoughts [14, 15].

With the outbreak of the COVID-19 pandemic, researchers found an increase in the proportion of depressive symptoms compared to the pre-pandemic times [11–13, 16]. For example, the results of a repeated cross-sectional study among Swiss students showed that 10.9% female and 8.5% male students reported depressive symptoms prior to the pandemic, whereas they reported significantly higher depressive symptoms during the pandemic, i.e., 30.8% female and 24.8% male students [16]. In addition, research suggested there was an increase in the prevalence of suicidal ideation among students as well [14, 15, 17]. Despite these concerning results, no drastic change in suicide rates during the COVID-19 pandemic was reported. A study across 33 countries [18] reported no increase in suicide rates in most countries. Moreover, there was no detected increase in help-seeking behavior in young adults [10, 11].

### Risk and protective factors for suicidal ideation

To understand the apparent increase in mental health difficulties faced by students during this time, additional risk factors for mental health problems need to be explored. It has been suggested that the high prevalence of mental health difficulties among university students (as opposed to other demographic groups) is related to their exposure to numerous risk factors that can lead to poor mental health outcomes [19]. Regarding suicide, many risk factors have been identified, and can be divided into four levels: (i) Individual level (e.g., genetic and biological factors, personality characteristics, prior suicide attempt, mental disorders, chronic disease and pain); (ii) relationship level (e.g., social isolation, loss of important relationships, relationship conflict, lack of social support); (iii) community level (e.g., discrimination, natural disasters); and (iv) societal level (e.g., health system characteristics, lack of social security, social norms, attitudes) [20, 21]. Contemporary classifications emphasize the interaction between different levels of risk factors, as well as the temporal component of their effect on suicidal behavior. Risk factors are described as either distal, proximal or developmental, depending on when their effect on suicidal behavior occurs [21].

Understanding the impact of the COVID-19 pandemic on students’ mental health requires insight into protective factors as well. Two factors, which may act as protective factors against suicidality are resilience [22–27] and social support [28–30]. Existing research on these risk and protective factors is briefly summarized below.

#### Gender

The age-standardized suicide rate was globally 2.3 times higher in males than in females [2], whereas in Slovenia, men die by suicide three to four times more often than women on average, and this ratio increases with age [3]. Other data shows that suicidal ideation [15, 31], as well as attempts [32], might be more common in women. The COVID-19 pandemic appears to have particularly affected female tertiary students, who reported more suicidal ideation than males [33, 34]. Moreover, the systematic review of Martínez-Líbano and Cabrera [35] found female students to have a higher risk for suicidal ideation and male students to have a higher risk of completing suicide.

#### Pre-existing mental health-related problems

The association between suicide and mental disorders, in particular depression and alcohol use disorders, is well researched [1]. Nevertheless, by far the strongest risk factor for suicide is a previous suicide attempt [1]. Pre-existing mental health problems were also identified as a risk factor for suicidal ideation during the pandemic [35–37].

#### Relationship status and living arrangement

A study by Shaw and colleagues [38] reports that living alone or with a non-partner was correlated with suicide in men, while no such associations were found in women. Similarly, a meta-analysis [39] showed that in men, a non-married status was a significant risk factor for suicide. In women, this association was only reported for those younger than 65 years. Regarding differences in suicidal ideation, a study on young adults [40] reported that suicidal ideation did not vary based on relationship status (marriage, cohabitation, dating, and single status). A high-quality relationship was also a protective factor for suicidal ideation [40].

#### The quality of relationships in a household

One of the studies [31] showed that university students with suicidal ideation had similarities in their home life – poor family structure and relationships, parents’ unstable employment, and improper parenting style. Similarly, a study of college students [41] showed that suicidal ideation significantly correlated with higher mother-child conflict and father-child conflict.

#### Somatic factors

A study by Ferro and colleagues [42] reported that people aged 15 to 30 years with a chronic illness are more likely to report mood disorders and drug disorders (excluding alcohol and cannabis), while also having suicidal thoughts, plans and attempts more commonly than controls. A study by Wathelet et al. [34] confirmed relations between increased prevalence of suicidal ideation and chronic conditions as well.

#### Psychological resilience

Psychological resilience was proven to be exceptionally important for promoting students’ well-being during the COVID-19 crisis [26, 27]. It refers to individuals’ ability to retain good functioning despite exposure to stress or trauma [43], which was not an uncommon experience during the pandemic. Individuals significantly vary in their response to stress – while some people who experience prolonged stress develop stress-related psychiatric disorders (such as depression), the majority of individuals exposed to stress manage to maintain normal psychological functioning [44]. Han and Nestler [44] also proposed neural mechanisms of susceptibility versus resilience to depression, which have already been tested in animal models. Resilience was found to be negatively associated with depressive symptoms [25], suicidal ideations [22–24], interpersonal difficulties and learning problems [45].

#### Social support

Another protective factor, which represents a resource for dealing with difficult situations, is social support. Its importance as a mental health factor became especially apparent during the COVID-19 pandemic, when socializing was hindered due to government-imposed restrictions. Many students reported increased levels of worry due to poorer social support during this period [29]. According to Kleinman and Liu [32], social support is associated with a decreased likelihood of a lifetime suicide attempt. Moreover, low levels of social support in students were associated with depressive symptoms [28] as well as suicidal ideation and thoughts [30, 41]. When studying the effect of different facets of social support, Arenson and colleagues [46] found that social network size predicts lower suicidal ideation, while perceived social support does not. Aim of the study.

**Fig. 1:**
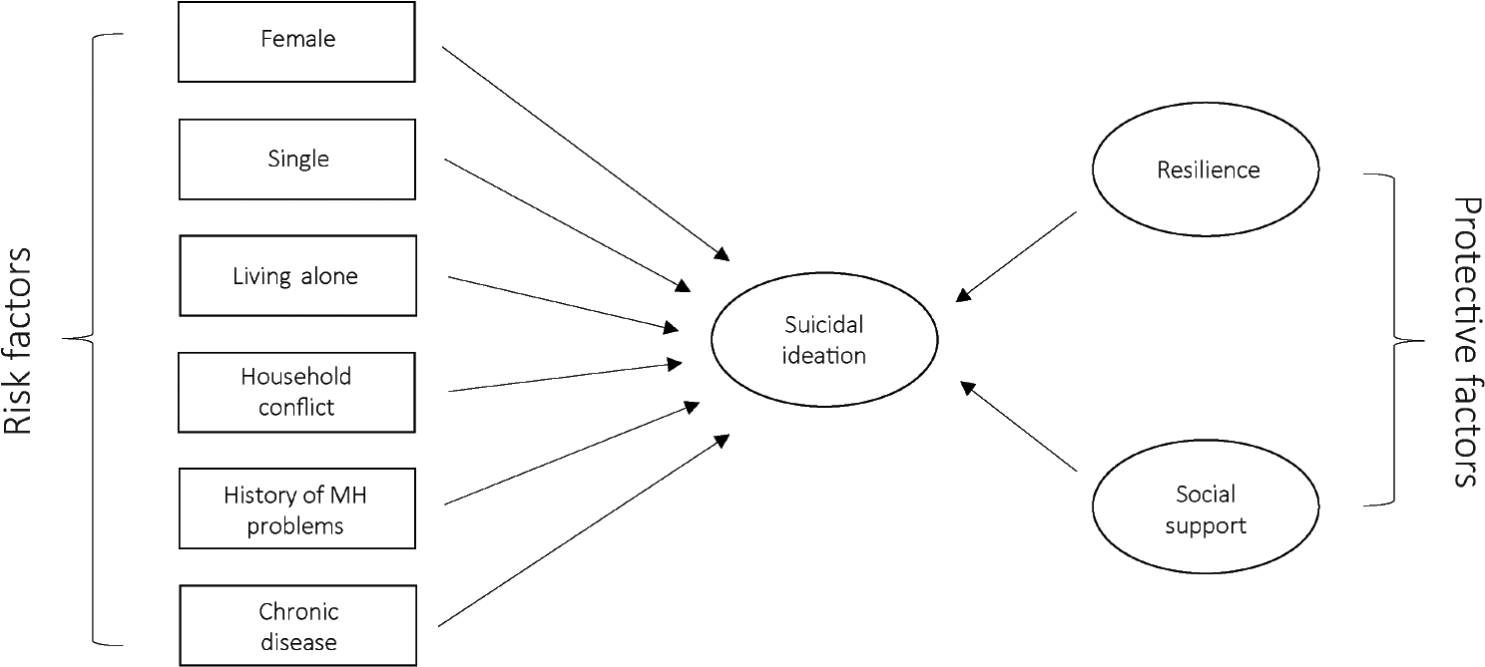
Proposed model of risk and protective factors of suicidal ideation

Considerable research on the role of certain factors in depression symptoms and suicidal ideation exists, suggesting the predictive potential of many variables. However, much less is clear regarding the interaction between the various risk and protective factors. In particular, to our knowledge, no study has assessed to what extent can resilience and/or social support mitigate the harmful effects of various life circumstances (especially those related to the COVID-19 pandemic) on depression symptoms and/or suicidal ideation. Thus, the aim of the study was twofold. Firstly, the goal was to examine whether selected measures of living conditions quality (living arrangement, household conflict, relationship status) and personal circumstances (gender, history of mental illness, having a chronic disease) predict the presence of depression symptoms, and suicidal ideation in particular, among tertiary students. Secondly, the aim was to examine how resilience and social support contribute to the likelihood of developing depression symptoms, as well as suicidal ideation among tertiary students, and to what extent can resilience and social support mitigate the effects of difficult life circumstances (see Fig. 1).

## Methods

### Sample

The population in the present study were Slovenian tertiary students (both university students and students of other institutions offering post-secondary education) who were enrolled in the study year of 2020/2021 and were fluent in Slovene. The sample consists of 4,645 tertiary students, representing 77% of the 5,999 participants who provided complete responses to the relevant questions. 73.3% of the sample identified as women (*n* = 3,403), 26.7% as men (*n* = 1,242). The average age of participants was 22.9 years (SD = 3.2), ranging from 18 to 57 years. More than half were not in a relationship (55.5%, *n* = 2,577), whereas only 3.0% (*n* = 139) were living alone at the time. Lastly, 17.9% (*n* = 833) of students were suffering from a chronic disease, and 27.6% had previously been diagnosed with a mental disorder (*n* = 1,280). According to the data provided by the Slovenian statistical office [47, 48], the number of full-time tertiary students in Slovenia in the same study year was 82,694; 34,992 men (42.4%) and 47,702 women (57.6%). Thus, the present sample represents around 6% of the entire population.

### Procedure

#### Data collection

The presented data was collected as a part of a large national cross-sectional online study assessing various aspects of mental health and its factors in tertiary students during the COVID-19 pandemic. To reach as many tertiary students as possible, all Slovenian universities, private faculties and student organizations were invited to participate, and to forward the link to the online survey to all their students. The invited institutions largely responded by posting the invitation on their websites and social media platforms, as well as by sending e-mails to their students. At the beginning of the survey, participants were informed about the aim of the study and their rights. Specifically, they were informed of their right to withdraw from the study at any time without consequences, that their data would be handled in accordance with European and Slovenian laws on personal data protection, that their anonymity would be ensured, and that the provided data would be used for research purposes only. An email address for any additional questions regarding participation in the survey was also provided. Following this, participants were required to provide informed consent before proceeding to answer any survey questions. Data collection took place through a web-based survey from February 9th to March 8th, 2021.

#### Statistical analyses

The statistical methods used consisted of the following: (i) frequencies and descriptive statistics; (ii) assumptions testing for performing a multiple linear regression: independence of observations, linearity of relationships, normality of residuals, no extreme outliers, absence of multicollinearity and homoscedasticity; (iii) assumptions testing for performing a binary logistic regression: independence of observations, absence of multicollinearity, no extreme outliers, and linearity of the relationship between predictor and the logit of the response variable; (iv) hierarchical multiple linear regression to evaluate the contributions of various predictor variables towards depression scores, and to obtain and compare the variance explained (R^2^) by the two groups of factors (life circumstance variables in the first group, with the addition of social support and resilience in the second group); and (v) hierarchical logistic regression to evaluate the contributions of various predictor variables towards the presence of suicidal ideation, and to compare the improvement of model fit by adding each of the two groups of predictor variables.

In the case of the regression model with depression scores as the outcome, there were only minor discrepancies from the assumptions of linearity, normality of residuals, and homoscedasticity, while conditions regarding independence, multicollinearity and the absence of extreme outliers were satisfied. Therefore, we proceeded with hierarchical multiple linear regression. However, in the case of suicidal ideation as the outcome of the regression model, we observed that the conditions of linearity and normality of residuals, in particular, were not met. Therefore, we re-coded the suicidal ideation variable to a dichotomous one (i.e., suicidal ideation present versus not present), and performed a hierarchical logistic regression. We did not find any major violations of its respective assumptions (listed above).

The data of the students who did not provide complete responses to our variables (23% out of 5,999) primarily consisted of those who begun filling out the survey but then quit prematurely. Since large portions of their data were missing anyway, those data units were list-wise deleted and excluded from the final sample. All statistical analyses were performed using IBM SPSS Statistics (Version 25) [49].

### Measures

#### Depression symptoms

To provide an assessment of our two study outcomes, we used the Patient Health Questionnaire (PHQ-9; [50]), an instrument assessing depression symptoms during the last 14 days. The PHQ-9 is a measure of depressive symptoms and consists of nine items in the following format: “Over the last 2 weeks, how often have you been bothered by any of the following problems?” The severity of various issues is reported on the scale: 0 = Not at all, 1 = Several days, 2 = More than half the days, 3 = Nearly every day. Depression symptoms score was calculated as the sum across all nine items. The Slovenian PHQ-9 has shown adequate psychometric characteristics (e.g. [51]).

#### Suicidal ideation

Item 9 of the PHQ-9 specifically evaluates passive thoughts of death or self-injury within the last two weeks [52]. Respondents report the presence of “Thoughts that you would be better off dead or of hurting yourself”. It is often used to screen depressed patients for suicide risk, and it was shown to correlate with other scales assessing suicidality [52]. Previous research [9, 53] suggests suicidal ideation measured by the 9th item of the PHQ-9 may function as a robust predictor of suicide attempts and deaths.

#### Social support

The Oslo Social Support Scale (OSSS-3; [54]) is a brief measure of social support, consisting of three items that ask for the number of close confidants, the sense of concern from other people, and the relationship with neighbors with a focus on the accessibility of practical help. The scale demonstrates adequate construct (i.e., one latent factor) and predictive validity (association with several health and well-being related outcomes) [55]. Scores range from 3 to 14, with higher scores indicating strong social support and lower scores indicating poorer support.

#### Resilience

Resilience was assessed with the Connor–Davidson Resilience Scale (CD-RISC-10) [56], i.e., the 10-item version adapted by Cambell-Sills and Stein [57], which was shown to have adequate internal consistency and construct validity. Respondents report their agreement with ten statements on a 5-point Likert Scale from “Not true at all” to “True nearly all the time”. A higher degree of agreement indicates greater resilience of the individual. The Slovenian CD-RISC-10 showed adequate reliability and construct validity [58].

#### Other measures

Other measures included in the present study due to their potential of predicting depression symptoms were gender (men/women), relationship status (single/in a relationship), living arrangement (living by yourself/living with others), degree of conflict within the household (on a 7-point scale from “No-conflict at all” to “A lot of conflict”), history of mental illness (yes/no), and presence of chronic diseases (no chronic disease/one or more chronic disease).

## Results

### Descriptive statistics

Basic descriptive statistics (and Cronbach alphas) of variables used in analysis are available in Table 1.

**Table 1:** Frequencies, means, standard deviations, ranges and Cronbach alphas for relevant variables

Variable	Category	%	Variable	Mean (SD)	Min	Max	Cronbach alpha
Gender	Male	26.9	Household conflict	2.55 (1.23)	1.0	7.0	-
	Female	73.1					
Relationship	Single	55.1	Social support	9.65 (2.25)	3.0	14.0	0.577
	Relationship	44.9					
Living arrangement	Alone	3.3	Resilience	23.67 (7.36)	0.0	40.0	0.885
	With others	96.7					
Chronic disease	Yes	17.5	Depression symptoms	20.20 (7.24)	9.0	36.0	0.908
	No	82.5					
History of mental illness	Yes	29.2					
	No	70.8					
Suicidal ideation	Yes	26.6					
	No	73.4					

### Predicting depression scores

The hierarchical regression model of depression scores consisted of two groups (or blocks) of predictors. The first block consisted of variables describing various life circumstances; gender (women vs. men), relationship (single vs. in a relationship), living arrangement (living alone vs. living with others), degree of household conflict, history of mental illness (yes/no) and presence of chronic diseases (yes/no). Taken together, these variables explained 14.8% of the variance in students’ depression scores. Table 2 shows each of the listed variables as a significant predictor of depression scores; being a woman, single, living alone, with higher household conflict, having a history of mental illness and being diagnosed with any chronic disease are associated with higher depression scores. The degree of household conflict and history of mental illness stand out as the strongest predictors of depression scores in this group.

A significant improvement in model fit (*F*(2,4674) = 566.85, *p* < .001) was found after inserting the second block of predictor variables into the model (i.e., Model 2). The second block consisted of social support and individuals’ resilience. Taken together, the predictor variables now account for 31.5% of variance in depression scores, which represents an increase of 16.7% points compared to the previous model. Table 2 reveals that social support and resilience are both strong and statistically significant predictors of (low) depression score. Particularly resilience, however, has emerged as the strongest of all predictors of depression score. Simultaneously, the predictive power of household conflict and history of mental illness, the two strongest predictors in Model 1, has decreased significantly in Model 2.

**Table 2:** Regression coefficients, statistical significance and collinearity statistics of predictors in model 1 and model 2. Outcome: Depression symptoms

Model			Unstandardized coefficients		Standardized coefficients		Sig.	Collinearity statistics	
	R^2^	Variable	*B*	Std. error	Beta	*t*		Tolerance	VIF
1	0.148	(Constant)	25.975	1.216		21.353	0.000		
		Gender	-1.882	0.225	− 0.115	-8.382	0.000	0.970	1.031
		Relationship	-1.072	0.200	− 0.074	-5.361	0.000	0.971	1.030
		Living arrangement	-3.252	0.592	− 0.077	-5.489	0.000	0.937	1.067
		Household conflict	1.331	0.083	0.226	16.077	0.000	0.929	1.077
		History of mental illness	3.295	0.226	0.204	14.583	0.000	0.941	1.063
		Chronic disease	1.868	0.261	0.099	7.167	0.000	0.958	1.044
2	0.315	(Constant)	37.347	1.162		32.129	0.000		
		Gender	-1.124	0.205	− 0.069	-5.486	0.000	0.937	1.067
		Relationship	− 0.707	0.180	− 0.049	-3.932	0.000	0.966	1.036
		Living arrangement	-2.575	0.533	− 0.061	-4.832	0.000	0.932	1.073
		Household conflict	0.804	0.077	0.136	10.492	0.000	0.872	1.147
		History of mental illness	2.198	0.205	0.136	10.710	0.000	0.917	1.091
		Chronic disease	1.357	0.234	0.072	5.795	0.000	0.954	1.048
		Social support	− 0.379	0.043	− 0.118	-8.894	0.000	0.838	1.194
		Resilience	− 0.372	0.013	− 0.378	-28.415	0.000	0.832	1.201

### Predicting suicidal ideation

The hierarchical logistic regression model of suicidal ideation consisted of the same two blocks of variables as above. Similar to before, inserting both blocks of variables significantly improved the predictive model, as shown by the Omnibus tests of model coefficients (χ^2^(6) = 365.23, χ^2^(2) = 518.15; *p* < .001). However, according to the Hosmer and Lemeshow test, Model 1 still shows a relatively poor model fit (*p* = .015), indicating that the life circumstances variables fail to sufficiently explain suicidal ideation. Indeed, Model 1 achieves correct classification (suicidal thoughts present vs. non-present) in only 73.7% of cases, which is only a marginal improvement of accuracy compared to the Intercept (null) model (73.0% accuracy).

Looking at the variable level (Table 3), all but gender are statistically significant predictors of suicidal ideation. The odds ratio (Exp(B) in Table 3) reveals that having been diagnosed with a mental illness at any point in life makes suicidal ideation at least two times more likely on average. Additionally, an increase of one point on a seven-point household conflict scale makes suicidal ideation 38% more likely. Living alone, having a chronic disease and being single correspond to a higher likelihood of suicidal thoughts as well, although not as substantially.

**Table 3:** Coefficients given by binary logistic regression for model 1 variables. Outcome: suicidal ideation

		B	S.E.	Wald	df	Sig.	Exp(B)
Model 1	Gender	− 0.151	0.080	3.521	1	0.061	0.860
	Relationship	− 0.343	0.071	23.483	1	0.000	0.710
	Living arrangement	− 0.809	0.191	17.930	1	0.000	0.445
	Household conflict	0.320	0.028	131.063	1	0.000	1.377
	Chronic disease	0.396	0.085	21.508	1	0.000	1.486
	History of mental illness	0.768	0.074	108.180	1	0.000	2.156
	Constant	0.105	0.394	0.071	1	0.791	1.110
Model 2	Gender	0.003	0.087	0.001	1	0.972	1.003
	Relationship	− 0.281	0.076	13.822	1	0.000	0.755
	Living arrangement	− 0.743	0.202	13.573	1	0.000	0.475
	Household conflict	0.213	0.030	48.762	1	0.000	1.237
	Chronic disease	0.297	0.092	10.491	1	0.001	1.346
	History of mental illness	0.550	0.079	48.066	1	0.000	1.733
	Social support	− 0.147	0.017	71.333	1	0.000	0.863
	Resilience	− 0.097	0.006	291.362	1	0.000	0.908
	Constant	3.608	0.454	63.137	1	0.000	36.876

After inserting the second block of variables into the predictive model, we notice that the model fit significantly improves, as shown by the Hosmer and Lemeshow test (*p* = .612). Additionally, the classification accuracy of the model has increased to 76.9%. Both social support and resilience significantly predict the likelihood of suicidal ideation, where higher scores translate to a lower likelihood of suicidal ideation (Table 3). Importantly, the odds ratio statistics for life circumstances predictors are now noticeably lower, particularly in the case of mental illness history and gender, with the latter having next to no predictive power in the context of Model 2. On the other hand, the effect of living alone on the likelihood of suicidal ideation has remained largely unaffected and persists even after the addition of social support and resilience into the model.

## Discussion

During the COVID-19 pandemic, tertiary students were faced with a variety of novel challenges associated with the shift towards online education and the prolonged restrictions regarding socializing, leisure activities, travelling, visiting family, etc. We expected that the quality of living conditions and certain personal circumstances in this period would become crucial risk factors for students’ depression symptoms and suicidal ideation. The collected data certainly supported this hypothesis, since each of the measured living conditions characteristics (living arrangement, household conflict, relationship status) and personal circumstances (gender, history of mental illness, chronic disease) significantly predicted the presence of depression symptoms, collectively explaining close to 15% of depression score variance.

When examining the effect of living conditions and personal circumstances on suicidal ideation, we found significant effects in all variables except for gender. Whereas women students were more prone to experiencing depression symptoms during the pandemic, they did not necessarily experience more suicidal ideation, contrary to previous research [15, 31]. Moreover, the model fit was found to be suboptimal, indicating that suicidal ideation could not be sufficiently explained by students’ living conditions and personal circumstances during the pandemic. Among the predictor variables describing life circumstances, commonly experiencing conflict at home and having been diagnosed with any mental illness in the past were the strongest predictors of depression symptoms and suicidal ideation specifically. The importance of non-hostile relationships at home for one’s general mental health was in line with our expectations, while the extent of its effect on suicidal ideation, a quite specific indicator of mental health, was quite remarkable, although consistent with some of the prior work on adolescents (e.g. [58–60]).

In the second stage of both hierarchical regression models, we aimed to explore the role of social support and resilience, two variables representing one’s resources and capacity to persevere in challenging situations. We expected that these would function as protective factors against mental health issues, mitigating the risks associated with difficult life circumstances. Indeed, introducing social support and resilience into the predictive model of depression symptoms more than doubled the variance explained by the previous group of variables. In the case of suicidal ideation, the addition of social support and resilience into the model significantly improved the model fit and increased the chance of correctly classifying a case as either symptomatic or not by more than 3%. Accounting for one’s social support and resilience also noticeably reduced the predictive effect of household conflict and history of mental illness on depression symptoms and suicidal ideation. The role of these two concepts offers an explanation of why some individuals manage to maintain mental well-being and stability in difficult life circumstances, while others in similar situations develop mental health issues and can even become suicidal.

The statistical significance of social support as a predictor of depression symptoms and suicidal ideation indicates that students with similar life circumstances (e.g., single, living alone) can experience markedly different levels of social support, which in turn translates to a different likelihood of feeling depressed or suicidal. We propose one of the key factors of having social support during the pandemic was the capability of having fulfilling interpersonal interactions online and thus maintaining high-quality relationships with family and friends despite physical limitations. These findings confirm the role of social support as a protector against suicidal ideation of college students, as found in previous studies [61–63].

Finally, an individual’s resilience, i.e., the ability to endure, persevere or even thrive during difficult events or life periods (e.g., [43]), showed the strongest effect on depression symptoms and suicidal ideation. Students who self-assessed to be more resilient in general were much less likely to report experiencing depression symptoms in the past two weeks. Moreover, gaining a single point on the 40-point resilience scale translated to a 9% decrease in the likelihood of suicidal ideation. These findings are consistent with previous work on the effect of resilience on suicidal ideation in college students [23, 24] and add to the range of contexts in which its protective function against depression symptoms has been documented (e.g. [64–67]).

The extent of the variance in depression symptoms, which resilience and social support account for, reveals the importance of these protective factors and their potential to overpower the effect of many harsh circumstances in life, such as having a chronic disease, a history of mental disorders, or regularly experiencing conflict at home. It seems paramount to start exploring multiple avenues towards promoting and increasing the resilience and social support of the general population since these personal qualities will likely be among the most effective means of preventing mental illness. Moreover, we should work on developing programs aimed at establishing and/or improving the conditions for developing resilience and social support in all vulnerable populations. Ideally, these programs would include systematic (for example, school-based) interventions, and would primarily focus on children or adolescents, and eventually start targeting the adult population as well. These programs should rely on peer-reviewed research, such as case studies, randomized-control trials and cohort studies on how resilience or social support can be developed over time.

While attempting to increase resilience and social support would be beneficial, the responsibility for maintaining adequate mental health should not be placed entirely on individuals. The path towards better mental health also consists of minimizing the difficult circumstances that individuals have to endure. Hopefully, in the case of the COVID-19 pandemic, we have learned some valuable experience as a society and especially decision-makers on how to establish appropriate measures which would take both mental and physical health of citizens into account [67, 68]. Since the results show the vital role of social support in protecting individuals from depression symptoms and suicidal ideation, it was important not to obstruct the conditions for social support (e.g., social interaction in person), which effectively introduced a significant risk for mental health problems in many individuals.

### Limitations

An important limitation of the study is associated with missing data (i.e., non-complete responses) due to participants quitting the online survey. Likely, the missing data was not missing completely at random, which introduces the possibility of bias in the results. Additionally, the sample contains a disproportionate number of women compared to men, which indicates room for improvement in sample representativeness. Considering the sample size is still relatively large (as a proportion of the entire population), we expect that the population parameters (i.e., regression coefficients) were, nevertheless, assessed with reasonable accuracy. Finally, suicidal ideation was measured with only one self-reported item. A significant contribution would be the inclusion of a more comprehensive measure of suicidal ideation, such as the lexicon-based approach [69] or the machine learning algorithm approach [70], which have the added benefit of being less subjective.

## Conclusion

The quality of students’ living conditions and personal circumstances during the COVID-19 pandemic importantly determined their risk for experiencing depression symptoms and suicidal ideation. In particular, students experiencing conflict at home and those with a history of mental illness were subjected to the most risk for suicidal ideation. However, resilience and social support proved to function as crucial protective factors against both depression symptoms and suicidal ideation, with resilience being found to be the most powerful predictor of students’ suicidal ideation among all of the variables included in the present study. Moreover, this underscores the importance of not only minimizing risk factors but also strengthening protective factors to mitigate the negative impact of potential future crisis situations on mental health.This valuable insight may be used in preventive efforts against this public health issue to inform the design and implementation of targeted interventions. Future research should further explore to what extent resilience (and social support) can protect against other mental health issues in contexts beyond the pandemic and other populations.

## Data Availability

The dataset generated and analysed during this study is available upon reasonable request from the corresponding author.

## Abbreviations

PHQ-9: Patient Health Questionnaire
OSSS-3: The Oslo Social support scale
CD-RISC-10: Connor-Davidson Resilience Scale
